# ECI Biocommentary: Olga Romantsik

**DOI:** 10.1038/s41390-022-02156-y

**Published:** 2022-06-21

**Authors:** O. Romantsik

**Affiliations:** Department of Clinical Sciences Lund, Division of Pediatrics, Lund University, Skåne University Hospital, 222 42 Lund, Sweden


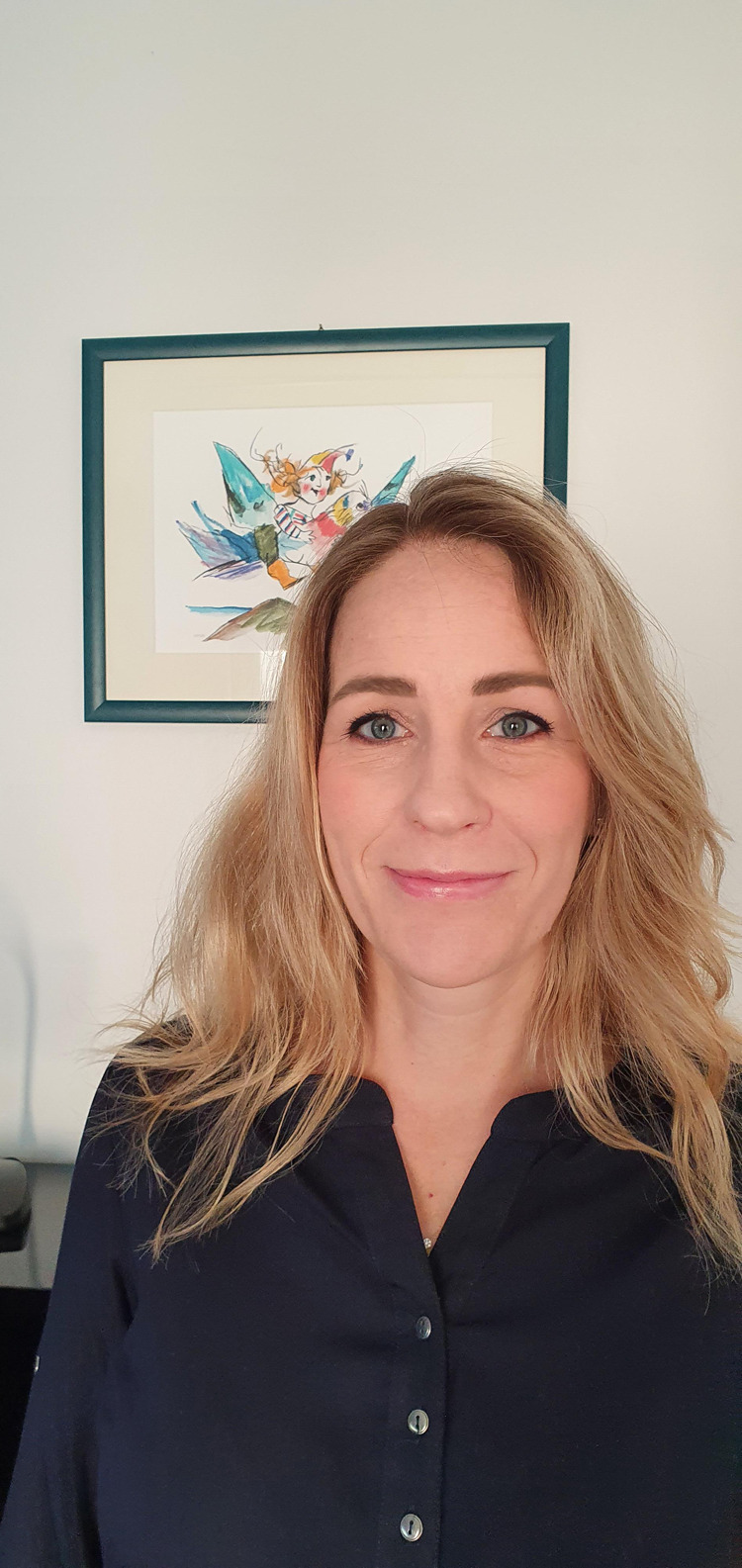


I was born and grew up in Tallinn, Estonia. I completed my medical studies at Tartu University, which was founded in 1632. When I was in medical school, I met Professor Heili Varendi who introduced me to the world of neonatology. She was pivotal in my choice to become a neonatologist. Indeed, I did my first research project on neonatal olfaction under her supervision. After my medical studies, I moved to Italy, where I completed my residency in Pediatrics and started my fellowship in Neonatology at Genoa University Hospital.

I have always been fascinated by brain development, both in health and disease. I became particularly interested in the disease of intraventricular hemorrhage (IVH). Facing patients with IVH and their families and knowing that IVH is not treatable became a driving force for my research. It brought me to Lund University, where I met Professor David Ley, who became my supervisor. Under his and Professor Xiaoyang Wang’s guidance, I started my Ph.D. project focusing on IVH pathophysiological mechanisms and potential treatment strategies. I was awarded funding from the ALF Swedish governmental grant and fulfilled my Ph.D. in March 2021. While working on the project, I was fortunate to meet Professor Bobbi Fleiss, who introduced me to the field of cortical development, and even though we have never met physically, I have learned a lot from her. In the study presented in this article, we show that severe IVH alters cerebral white and gray matter development.^1^

Following my first contact with Cochrane Collaboration, particularly Professor Roger Soll, I expanded my research field and became an author of several Cochrane reviews in neonatology. I believe that Cochrane reviews help clinicians to make the best treatment decisions for the patients.

Currently, I am working as a Consultant in Neonatology at Skåne University Hospital. I am grateful to all my mentors and people who came across my path, especially the tiny patients and their families. My motto is “No problem is insurmountable; with a little courage, teamwork, and determination a person can overcome anything” (LM Alcott).

My advice to those interested in research is to find a mentor who cares about your professional and personal growth. Do not be afraid to step out of your comfort zone, take every opportunity to learn new skills, it will enrich you. Create a relationship with your patients, it will keep your passion up to continue with your research.
